# Investigating the physical and electrical properties of La_2_O_3_ via annealing of La(OH)_3_

**DOI:** 10.1038/s41598-024-57848-8

**Published:** 2024-04-02

**Authors:** Walid Ismail, Aya Belal, Walied Abdo, Abdelhamid El-Shaer

**Affiliations:** 1 Physics Department, Faculty of Science, Kafrelsheikh University, Kafrelsheikh, 33516 Egypt; 2 Department of Pathology and Clinical Pathology, Faculty of Veterinary Medicine, Kafrelsheikh University, Kafr El-Sheikh, 33516 Egypt

**Keywords:** Lanthanum oxide (La_2_O_3_), Nanopowder, Hydrothermal method, Annealing temperature, XRD, SEM, FTIR, Electrochemical impedance spectroscopy (EIS), Mott Schottky, Materials science, Physics

## Abstract

A simple technique was utilized to fabricate pure hexagonal La_2_O_3_ nanorods by utilizing lanthanum(III) nitrate hexahydrate (La(NO_3_)_3_·6H_2_O) and ammonia (NH_4_OH). The La_2_O_3_ nanoparticles were analyzed using XRD, TGA, Raman, SEM, FTIR, TEM, PL spectroscopy, and Mott–Schottky techniques. The XRD analysis confirmed the production of La(OH)_3_ nanorods under appropriate conditions, which were then successfully converted into La_2_O_2_CO_3_ and finally into La_2_O_3_ nanorods through annealing. The TGA analysis showed that the total weight loss was due to water evaporation and the dissolution of minimal moisture present in the environment. The FTIR analysis confirmed the presence of functional groups. The SEM analysis revealed changes in morphology. The TEM analysis to determine the particle size. The PL findings showed three emission peaks at 390, 520, and 698 nm due to interband transitions and defects in the samples. The Mott–Schottky analysis demonstrated that the flatband potential and acceptor density varied with annealing temperature, ranging from 1 to 1.2 V and 2 × 10^18^ to 1.4 × 10^19^ cm^−3^, respectively. Annealing at 1000 °C resulted in the lowest resistance to charge transfer (Rct).

## Introduction

There are various rare earth metal oxides including La_2_O_3_, Y_2_O_3_, CeO_2_, Sm_2_O_3_, Nd_2_O_3_, Eu_2_O_3_, Lu_2_O_3_, and Gd_2_O_3_^1^. La_2_O_3_ is a p-type semiconductor and has a hexagonal phase structure^2^. It appears as a white solid that is insoluble in water. However, it can dissolve in acidic solutions and convert into lanthanum hydroxide upon absorbing moisture from the air^3^. La_2_O_3_ is considered one of the most desirable oxide semiconductors due to its unique chemical and physical properties. It has a wide band gap of 5.5 eV, is thermally stable and non-toxic, and has a significant relative dielectric constant (K > 20)^4^. Because of these properties, La_2_O_3_ is used in potential applications like biosensors^5^, catalysts^6^, dielectric layers^7^, fuel cells^8^, gas sensors^9^, rechargeable batteries^10^, photoelectric conversion, and optical devices for measuring various body temperatures, and biomedical^11,12^. Various nanostructures, such as nanoneedles, nanobundles, nanorods, nanowires, nanosheets, and nanopowders of La_2_O_3_, have been obtained using different methods, including the hydrothermal method^13^, chemical precipitation method^14^, microwave method^15^, sol–gel method^16^, sonochemical method^17^, solvothermal method^18^, reverse micelles method^2^, arc discharge method^19^, thermal decomposition^6^, and laser deposition method^20^. Among these techniques, the hydrothermal method is the most effective one to fabricate La_2_O_3_ nanoparticles due to its low cost, environmentally friendly nature, good homogeneity, and simple methodology^21^. S Karthikeyan et al. Discovered that the ammonia source had significantly superior thermal, structural, and optical properties where La_2_O_3_ nanoparticles were synthesized using ammonia triethanolamine (TEA) and polyethylene glycol (PEG). In summary, the produced La_2_O_3_ nanoparticles could find usage in gas sensors to identify CO and CO_2_ as well as dielectric applications^2^. S. Karthikeyan et al. Used the reflux approach, lanthanum nitrate and urea were able to react to produce lanthanum oxide nanoparticles. These nanoparticles were calcined for one hour at 500 °C in their prepared state. Morphological investigations showed that the as-prepared La_2_O_3_ micro-plates had a thickness of 50 nm and that the plate-like structure broke into smaller La_2_O_3_ nanoparticles during the calcination process. For MOSFET applications, the pre-pare La_2_O_3_ structure may be utilized^22^. L. Wang et.al. prepared the La_2_O_3_ NPs catalyst using the precipitation method, and then calcined it at 600 °C. La_2_O_3_ NPs demonstrated increased catalytic activity in the reaction that transformed glycerol into glycerol carbonate and urea^23^. Qiuying Mu et al. In order to create lanthanum oxide carbonate (La_2_O_2_CO_3_) nanorods, lanthanum hydroxide (La(OH)_3_) nanorods were synthesized at 60 °C. Then, the nanorods were calcinated to 400 °C for two hours in a furnace. After calcining the La(OH)_3_ nanorods for two hours at 800 °C, pure La_2_O_3_ nanorods could be formed effectively^24^. Jun-Gill Kang et al. The hexagonal phase La(OH)_3_ nanowires and La_2_O_3_ microboards were effectively manufactured using a hydrothermal technique at 900 °C. On the other hand, hexagonal La_2_O_3_ is unstable in surrounding conditions. Due to the hygroscopic nature of La_2_O_3_, it progressively re-transforms into a hexagonal phase La(OH)3 via rehydroxylation^25^. Xing Wang et al. fabricated La_2_O_3_ films at different annealing temperatures of 400, 600, and 800 °C, for 60 s through atomic layer deposition. It was found that after 400 °C annealing temperatures, amorphous disordered structures of the film can be obtained. After being annealed at 600 and 800 °C, only weak crystalline planes such as hexagonal (101) appear, indicating the possibility of converting the 10 nm La_2_O_3_ film into complete crystallized. After being crystallized, the refractive index increases dramatically while the bandgap is slightly decreased^26^. The annealing temperature can improve the lattice structure and NPs characteristics in our work. In biological applications, the temperature-dependent crystal size is significant. In this work, La_2_O_3_ nanoparticles were synthesized via hydrothermal method at different annealing temperatures ranging from 500 and 1000 °C. The synthesized nanoparticles are intended to be used for biological applications. To investigate the effect of annealing temperature on the microstructures, morphology, optical and photoelectrochemical characteristics of La_2_O_3_ nanoparticles, various characterization techniques were employed.

## Experimental methods

La_2_O_3_ nanoparticles were synthesized using a hydrothermal technique. The chemical solutions used in the process included lanthanum (III) nitrate hexahydrate (La(NO_3_)_3_·6H_2_O; 99%; BDH) and ammonia (NH_4_OH; 32%; PIOCHEM, EGYPT). To begin, 0.1 M lanthanum nitrate was dissolved in 100 mL of distilled water (Milli-Q, 18MΩ cm) at 60 °C. The mixture was vigorously stirred until it became clear. Then, liquid ammonia was added slowly, drop by drop, to adjust its pH to 10. The solution was then left undisturbed to allow the precipitation to occur. The precipitate obtained was gathered and rinsed with water and ethanol to ensure no unreacted substances remained. The washed powder was then dried in an 80 °C hot air oven for an entire night. Afterward, the dry powder samples were collected and crushed using a mortar before analysis. The experiment was conducted at different annealing temperatures ranging from 500 to 1000 °C. The following analysis techniques were used: X-ray diffraction (Shimadzu), Thermo Gravimetric analysis (TGA- Setaram Themys one +) to determine the total weight loss, Raman spectra using a WITec alpha300 R system, and Fourier transform infrared FTIR with single beam (Nicolet.iS10-U.S.A.) to identify the functional groups present in the synthesized samples. Scanning electron microscopy (SEM) (JSM-651OLV) was employed to analyze the morphological properties. Transmission electron microscopy (TEM) images were taken by (JEOL 2010–200 kV). The optical characteristics of La_2_O_3_ nanoparticles were examined by PL with a Kimmon He-Cd laser of 325 nm (3.82 eV). The CHI660E electrochemical workstation with the help of 0.5 M Na_2_SO_4_ was utilized to make Mott–Schottky and EIS.

## Results and discussion

### Structural studies

**Figure 1:**
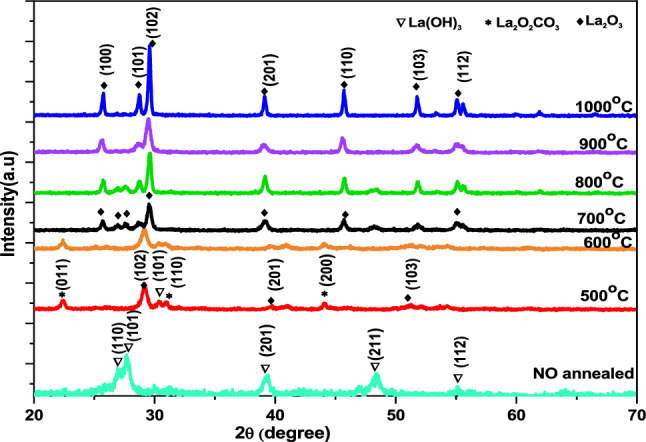
XRD patterns of La(OH)_3_, La_2_O_2_CO_3_ and La_2_O_3_ nanoparticles at different annealing temperatures.

Figure 1 shows XRD patterns for La_2_O_3_ NPs samples via annealing of La(OH)_3_. Samples fabricated at 60 °C, can be perfectly indexed to a pure hexagonal phase of La(OH)_3_ (JCPDS Card No. 13–1481) with lattice constant a = 6.528 Å, c = 3.858 Å and diffraction angles located at 2θ = 27°, 27.7°, 39.4°, 48.5° and 55° correlate with the (110), (101), (201), (211) and (112) planes^27^. La_2_O_2_CO_3_ and La_2_O_3_ can be produced via annealing of La(OH)_3_ at 500 °C and 600 °C for 3 h, respectively^28^. Therefore, samples annealed at 500 °C and 600 °C contain a mixture of La(OH)_3_, La_2_O_2_CO_3_ (JCPDS Card No. 37–0804) and La_2_O_3_ (JCPDS Card No. 73–2141) phase due to the partial transformation of La(OH)_3_. The diffraction peaks centered at 2θ = 23°, 32° and 44°, corresponding to (011), (110) and (200) planes, can be assigned to a hexagonal phase of La_2_O_2_CO_3_^24,29,30^. With the increase in annealing temperature until 1000 C^o^, La_2_O_2_CO_3_ is decomposed into La_2_O_3_ with hexagonal phase and lattice constants a = 3.973 Å, c = 6.129 Å. Diffraction angles related to La_2_O_3_ are located at 2θ = 26°, 28.6°, 29.9°, 39°, 46°, 52° and 55.3° and correspond with the (100), (101), (102), (201), (110), (103), and (112) planes, respectively^31^. Debye-Scherer’s relation was employed to estimate the average crystal size (D) by the following formula^32^.

1 $$ {\text{D}} = \frac{{0.9\uplambda }}{{\upbeta {\text{COS}}\uptheta }} $$

where D corresponded the crystal size, β is the FWHM indicated in radians, λ is X-ray wavelength and θ is the diffraction angle^33^.

It was observed that the crystallite size increased from 12 to 33.26 nm as the annealing temperature was raised. This could be attributed to two possible reasons; Firstly, higher temperatures may have facilitated ion diffusion in the samples. Secondly, the reduction of defects in the samples could have lowered the barriers between the particles, leading to the production of larger particles^34–38^. In order to determine microstrain (ε), the following equation is utilized^39^:

2 $$\upvarepsilon = \frac{{\upbeta \cot\uptheta }}{4} $$

The density of dislocation (δ) is represented by the following Eq. ^40^:

3 $$\updelta = \frac{1}{{{\text{D}}^{2} }} $$

**Figure 2:**
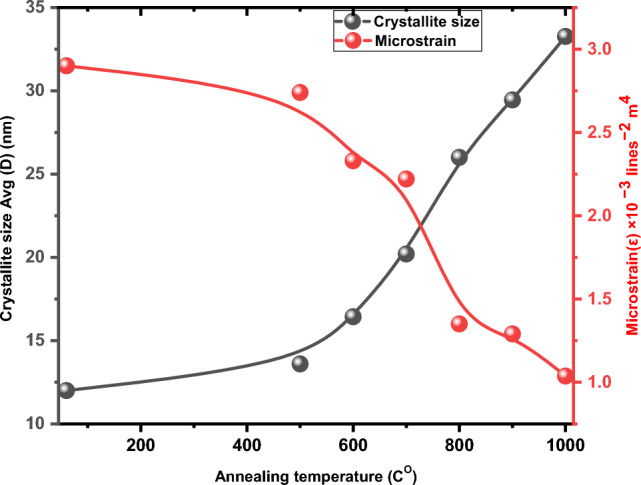
Variation of strain and crystallite size for La(OH)_3_, La_2_O_2_CO_3_ and La_2_O_3_ nanoparticles.

The values for microstructural properties are summarized in Table 1. Figure 2 displays that annealing at a higher temperature leads to fewer surface defects compared to non-annealed samples^41^.

**Table 1:** Structural parameter of Lanthanum Oxide at different annealing temperatures.

Simples Nanoparticles	Crystallite size Avg (D) (nm)	Dislocation density	Micro strain (ε) × 10^−3^ lines^−2^ m^4^
No annealing	12	6.94 × 10^−3^	2.9
annealing at 500^O^ C	13.6	5.86 × 10^−3^	2.74
annealing at 600^O^ C	16.43	3.70 × 10^−3^	2.33
annealing at 700^O^ C	20.2	2.49 × 10^−3^	2.22
annealing at 800^O^ C	26	1.47 × 10^−3^	1.35
annealing at 900^O^ C	29.45	1.15 × 10^−3^	1.29
annealing at 1000^O^ C	33.26	9.07 × 10^−4^	1.037

### TGA analysis

**Figure 3:**
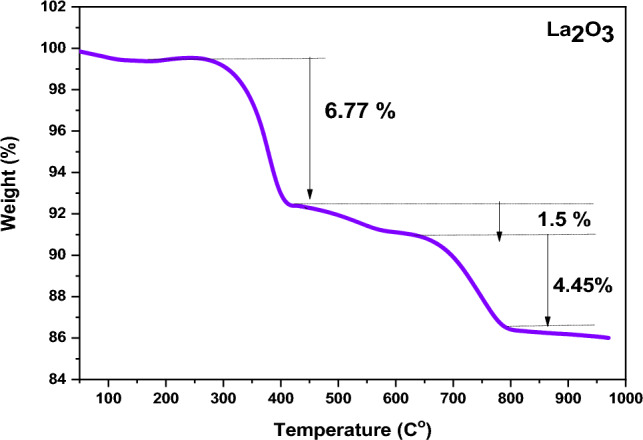
TGA curve of the dried powder at 60 °C.

To evaluate the La_2_O_3_ NPs' thermal stability, a Thermo Gravimetric analysis was performed in a nitrogen environment between room temperature and 1000 °C at a heating rate of 10 °C /min^42^. The TGA curve for the La_2_O_3_ NPs produced under nitrogen environment up to 1000 °C is shown in Fig. 3.

At a temperature of 100 °C, weight loss occurred in the low-temperature region due to the reduction of water molecules^43^. There was also a sharp weight loss observed at around 400 °C, indicating an endothermic process. The weight loss observed is related to the loss of carbon dioxide, and it amounts to approximately 6.77%^2,44^. At the high-temperature area, the breakdown of lanthanum carbonate and weight loss is approximately 1.5%. Finally, at temperatures above 700 °C, lanthanum oxy-carbonate dissolves and produces pure La_2_O_3_ nanoparticles^45^. It has been observed that the total weight loss of La_2_O_3_ NPs is caused by the evaporation of water and the dissolution of minimal moisture present in the particles. From the TGA curve, it has been estimated that only 12.77% of the total weight loss occurs. These findings clearly demonstrate that ammonia, which is used as a reducing agent in the fabrication process of La_2_O_3_ NPs, is highly stable. The ammonia is entirely removed by the washing process of water and ethanol, and annealing at 500 °C is sufficient to remove any unprocessed ammonia and additional impurities^2^. The decomposition pathway of La (OH)_3_ is proposed as follows^24^:

4 $$ {\text{La }}\left( {{\text{OH}}} \right)_{{3}} + {\text{ CO}}_{{2}} \to {\text{ LaOHCO}}_{{3}} $$

5 $$ {\text{LaOHCO}}_{{3}} \to {\text{ La}}_{{2}} {\text{O}}\left( {{\text{CO}}_{{3}} } \right)_{{2}} \cdot {\text{xH}}_{{2}} {\text{O }} + {\text{ yH}}_{{2}} {\text{O}} $$

6 $$ {\text{La}}_{{2}} {\text{O}}\left( {{\text{CO}}_{{3}} } \right){2} \cdot {\text{xH}}_{{2}} {\text{O }} \to {\text{ La}}_{{2}} {\text{O}}\left( {{\text{CO}}_{{3}} } \right)_{{2}} + {\text{ xH}}_{{2}} {\text{O}} $$

7 $$ {\text{La}}_{{2}} {\text{O}}\left( {{\text{CO}}_{{3}} } \right)_{{2}} \to {\text{ La}}_{{2}} {\text{O}}_{{2}} {\text{CO}}_{{3}} + {\text{ CO}}_{{2}} $$

8 $$ {\text{La}}_{{2}} {\text{O}}_{{2}} {\text{CO}}_{{3}} \to {\text{ La}}_{{2}} {\text{O}}_{{3}} + {\text{ CO}}_{{2}} $$

### Raman analysis

**Figure 4:**
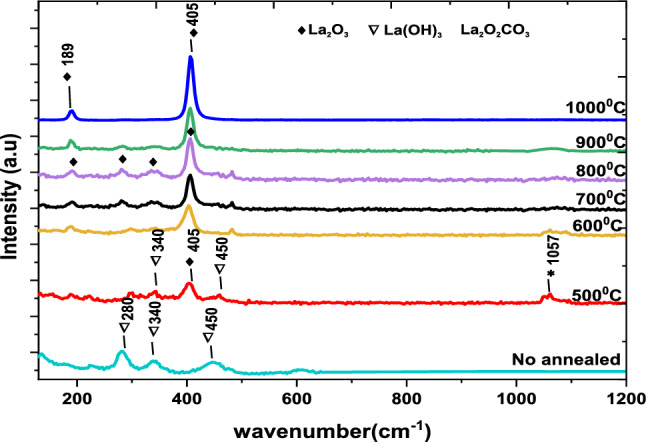
Raman spectrum of La(OH)_3_, La_2_O_2_CO_3_ and La_2_O_3_ at various annealing temperatures.

Raman spectra are used to confirm the results obtained from X-ray powder diffraction^46^. A laser Raman analysis was conducted to identify the phases, which ranged from 100 to 1300 cm^−1^, as shown in Fig. 4. At 60 °C, three vibrational modes were observed, which are related to only La(OH)_3_ phase^47^. These vibrational modes are A_1g_, E_2g_, and E_1g_ at 280, 340, and 450 cm^−1^, respectively. A_1g_ is a non-degenerate total-symmetric vibration, that means it is symmetric under all symmetry operations (rotation, mirror plane, inversion center). E_1g_, E_2g_ is a double-degenerate vibration that is symmetric under inversion^48^. By annealing at 500 °C and 600 °C, it was observed that four vibrational modes appeared around 340, 405, 450 and 1057 cm^−1^, which contained a mix of La (OH)_3_, La_2_O_2_CO_3_, and La_2_O_3_ phases^48^. With an increase in annealing temperature up to 1000 °C, vibrational modes were observed, which were related to only La_2_O_3_ phase^49^. It was found that the sharpness of Raman peaks increased with an increase in annealing temperature, which might be related to the increase in particle size^50^. These results are consistent with the XRD measurements.

### Fourier transform infrared spectroscopy analysis

**Figure 5:**
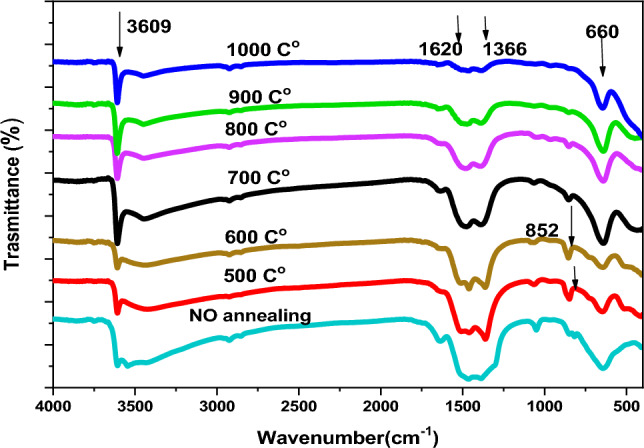
FTIR spectra of La(OH)_3_, La_2_O_2_CO_3_ and La_2_O_3_ at various annealing temperatures.

Figure 5 shows the FTIR spectrum of La_2_O_3_ NPs samples at different annealing temperatures, ranging from 4000 to 400 cm^−1^. The aim was to identify the functional groups present in the prepared samples^51^. The wide peak observed at 3609 cm^−1^ indicates the presence of O–H stretching vibration due to absorbed moisture on the nanoparticle surfaces^3,52^. The bands at 1620 cm^−1^ and 1366 cm^−1^ are caused by the O–H vibration in absorbed water on the sample surface^24,53^. The intensity of the peaks at 1620 cm^−1^ and 1366 cm^−1^ decreased with increasing annealing temperature. Two small peaks at 852 cm^−1^, observed at 500 °C and 600 °C, were related to C–O bending vibrations^2,16^. The broad absorption band measured at 660 cm^−1^ is thought to occur due to the La–O stretching vibration^24,54^. These bands demonstrated the existence of the La_2_O_3_ phase in the synthesized nanoparticles.

### Surface morphology evaluation

**Figure 6:**
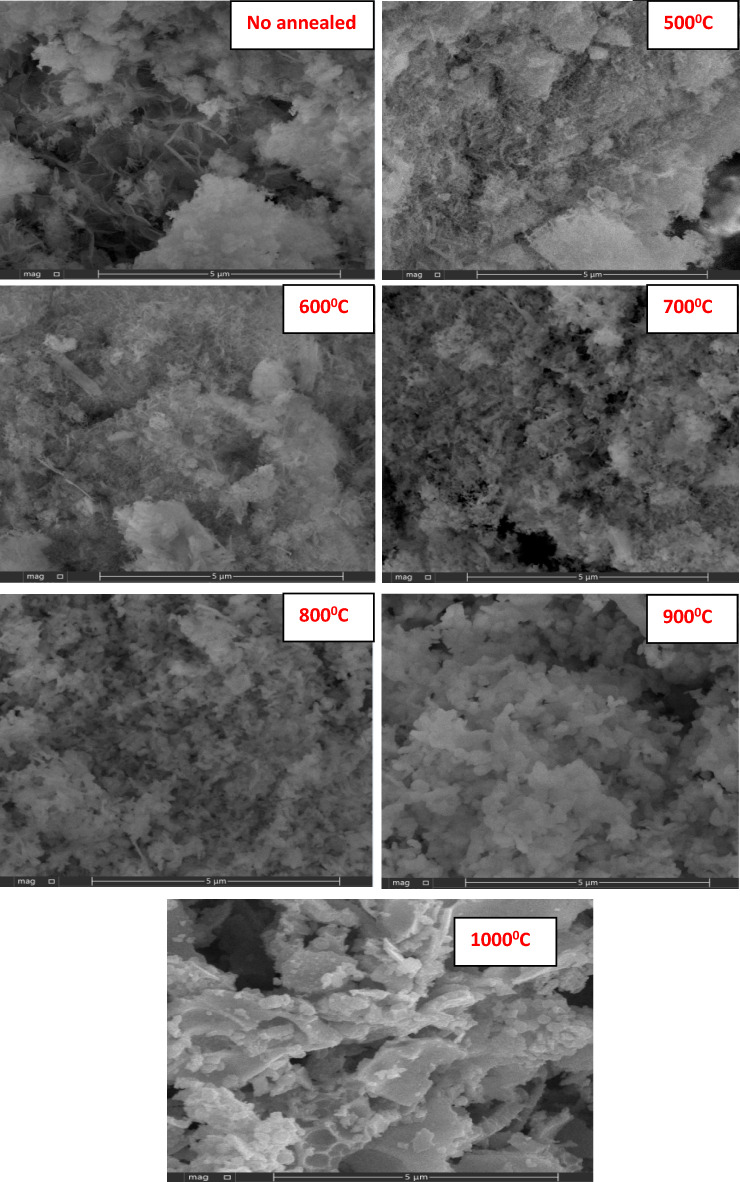
SEM photographs of La(OH)_3_, La_2_O_2_CO_3_ and La_2_O_3_ at various annealing temperatures.

The SEM technique was used to analyze the morphology of the La_2_O_3_ that was synthesized. Figure 6 The samples exhibit rod-shaped nanostructures with nanoparticle aggregation, which become more spherical at high temperatures, hindering size identification^24,55^. This aggregation could be influenced by annealing temperature that increases the mobility of atoms^52^. As the annealing temperature is increased, the nanoparticles' grain size also increases. The merging process is enhanced by the oxygen or lanthanum defects at the grain boundaries through the annealing of additional grains at high temperatures. It is proposed that grains with lower surface energy will expand more at higher temperatures. Moreover, higher temperatures provide more energy for the atoms to move and occupy their correct positions in the crystal lattice^35^. When the annealing temperature rises, this aggregation increases and produces large particles, as confirmed by XRD results.

### TEM pattern

**Figure 7:**
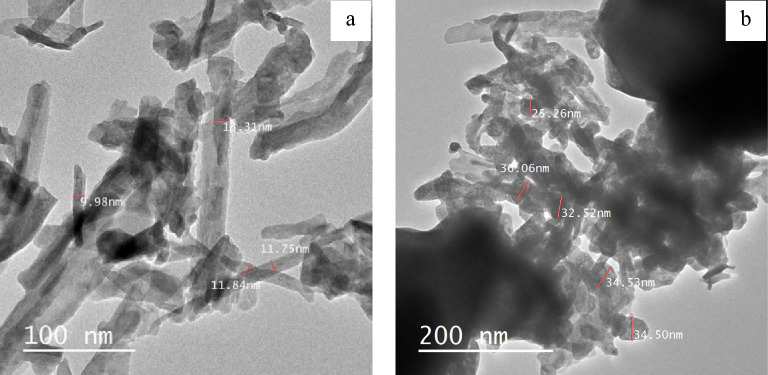
TEM images of **(a)** La_2_O_2_CO_3_ (annealed at 500 °C), and **(b)** La_2_O_3_ (annealed at 1000 °C).

Figure 7a,b, display TEM images of two samples, La_2_O_2_ CO_3_ and La_2_O_3_, that were fabricated and annealed at 500 °C and 1000 °C, respectively. The sample annealed at 500 °C showed aggregation and had nanorods with an average size of 11 ± 2 nm. As the annealing temperature increased to 1000 °C, the aggregation of particles increased which become more spherical at high temperatures with an average particle size of 31 ± 2 nm^56^. These findings agree with XRD results^57^.

### PL analysis

**Figure 8:**
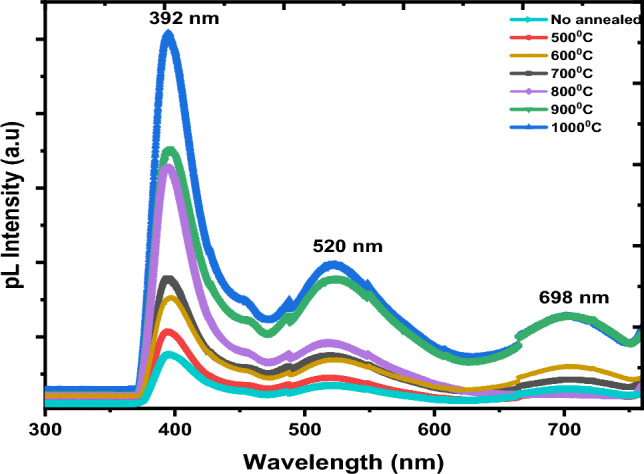
PL of La(OH)_3_, La_2_O_2_CO_3_ and La_2_O_3_ at different annealing temperatures.

The PL emission is caused by the recombination of free carriers. This makes PL spectra useful for analyzing the efficiency of trapped charges^58^. In Fig. 8, you can see the PL spectrum of all samples taken at room temperature with an excitation wavelength of 320 nm. The measurements were taken between 300 and 800 nm. The PL spectrum has three peaks: the first is around 390 nm, the second is located at 520 nm, and the third peak is found at 698 nm. The first peak is correlated with inter-band transitions in La_2_O_3_. The second peak is the result of cation vacancy defects in La_2_O_3_^59,60^. The third peak results from radiative relaxational transitions phenomena of La^3+^ ions^61^. It was observed that the peaks’ intensity increase with annealing temperature, showing that lanthanum vacancies may be enhanced by increasing of annealing temperature^61^.

### Electrochemical impedance spectroscopy (EIS)

**Figure 9:**
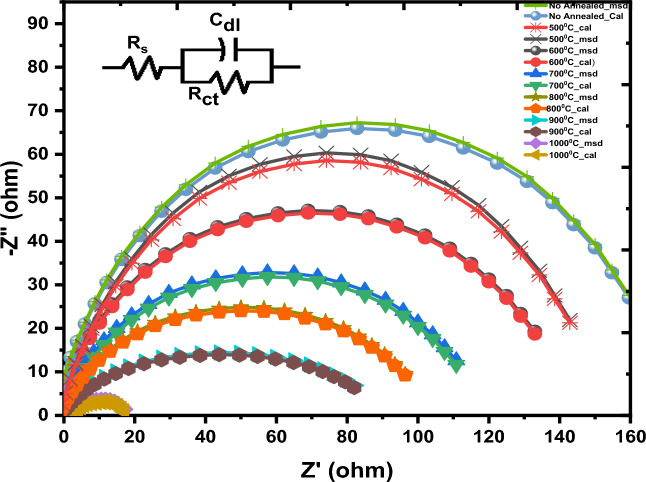
Nyquist plots and equivalent electrical circuit for La(OH)_3_, La_2_O_2_CO_3_ and La_2_O_3_ nanoparticles at various annealing temperatures.

The behavior of charge transfer at the interface between semiconductor and electrolyte was studied by examining the electrical responses of La_2_O_3_ nanoparticles using the electrochemical impedance spectra technique over a wide frequency range of 10^4^ HZ^62,63^. Figure 9 shows the Nyquist diagram of La_2_O_3_ nanoparticles, which were fabricated at various annealing temperatures ranging from 500 to 1000 °C. The Nyquist curves (Z" vs. Z') were fitted using ZSimpwing software )https://www.ameteksi.com/products/software/zsimpwin (and an electrical circuit model with the corresponding components of solution-resistance (Rs), charge-transfer resistance (RRCT), and double-layer capacitance (C_dl_)^64^. The plots are composed of semicircles, and their diameters decrease as the annealing temperature increases^41^. This suggests that there is a higher rate of charge transfer and a lower rate of charge recombination^65,66^. The reason for this is that when the annealing temperature increases, the electrons present in the valence band acquire enough energy to move into the conduction band. Because the number of electrons in the conduction band rose, conductivity is improved and resistivity declines^67^.

## Mott–Schottky measurements

**Figure 10:**
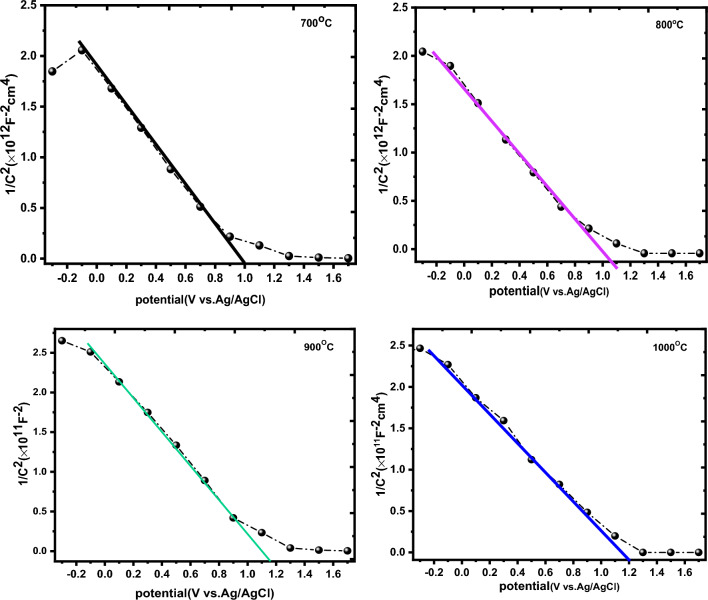
Mott–Schottky measurements for La_2_O_3_ nanoparticles at various annealing temperatures.

Mott–Schottky technique was used to determine the type of conductivity and compute the density of donors or acceptors in the fabricated samples^68^. The Mott–Schottky plot (1/C^2^ vs. V) is obtained from the electrochemical impedance data using the following relation^69^:

$$ {\text{C}} = \frac{1}{{2\uppi {\text{fXc}}}} $$

where f is 10 kHz and Xc represents the imaginary part (Z”). Figure 10 shows Mott–Schottky plots for the prepared La_2_O_3_ nanoparticles at various annealing temperatures. According to the Mott–Schottky formula, the acceptor density (N_A_), the semiconductor capacitance (C), and the built-in voltage (V_fb_) are related by the following formula^68^:

$$ \frac{1}{{{\text{C}}^{2} }} = \frac{2}{{ - \upvarepsilon \upvarepsilon _{0} {\text{eA}}^{2} {\text{N}}_{{\text{A}}} }}\left( {{\text{V}} - {\text{V}}_{{{\text{fb}}}} - \frac{{{\text{kT}}}}{{\text{e}}}} \right) $$

where ε,** ε**_**0,**_ e, k_B_, A, T represents dielectric constant (**ε** for La_2_O_3_ = 27), vacuum permittivity, charge of electron, Boltzmann constant, active surface area of the photoelectrode and temperature, respectively^70^.

V_fb_ could be obtained experimentally by interception of the linear part of the Mott–Schottky plot on the x-axis and it was observed to increase from 1 to 1.21 V with the raise in annealing temperature. The more the built-in voltage, the more the charge separation at the interface between the semiconductor and electrolyte, the acceptors density was estimated by the following formula^71^.

9 $$ {\text{N}}_{{\text{A}}} = \frac{2}{{\upvarepsilon \upvarepsilon _{0} {\text{eA}}^{2} {\text{S}} }} $$

where S represents the slope of the Mott–Schottky graph^70^. M-S plots exhibited a negative slope, showing that all samples were p-type semiconductors. The acceptors density improved from 1.3 × 10^17^ to 3.2 × 10^17^ cm^-3^ with the raise in annealing temperature, signifying a higher density of the lanthanum vacancies in La_2_O_3_ after it’s annealed at 1000 °C. Table 2 shows the calculated acceptors density and flat band potentials for La_2_O_3_ nanoparticles at different annealing temperatures.

**Table 2:** Effect of annealing on acceptors density and flat band potentials for La_2_O_3_ nanoparticles.

Samples	V_fb_ (V *vs*. Ag/AgCl)	Acceptors density (cm^−3^)
Annealing at 700 °C	1.14	2 × 10^18^
Annealing at 800 °C	1.17	2.4 × 10^18^
Annealing at 900 °C	1.2	1.2 × 10^19^
Annealing at 1000 °C	1.21	1.4 × 10^19^

## Conclusions

In this study, La_2_O_3_ nanoparticles were synthesized via hydrothermal method and characterized for microstructure, morphology, thermal, optical, and electrical properties. The XRD findings revealed that La(OH)_3_ nanorods were produced at 60 °C under appropriate conditions and could be converted into La_2_O_2_CO_3_ by annealing at temperatures of 500 °C and 600 °C for 3 h. Finally, the annealing temperature was raised until 1000 °C to obtain La_2_O_3_. According to TGA analysis, the weight loss observed in the manufactured La_2_O_3_ NPs was due to water evaporation and the dissolution of a minimal amount of moisture present in the environment. FTIR analysis confirmed the presence of functional groups related to La(OH)_3_, La_2_O_2_CO_3_, and La_2_O_3_. SEM results showed that the nanoparticles' aggregation increased with higher annealing temperatures. that the nanostructures were rod-shaped and became more spherical at high temperatures as the particle size increased. PL analysis revealed three peaks: the first peak was associated with inter-band transitions in La_2_O_3_, the second peak resulted from cation vacancy defects in La_2_O_3_, and the third peak resulted from radiative relaxational transitions phenomena of La^3+^ ions. The Mott–Schottky test results show that the flatband potential and acceptor density change depending on the annealing temperature. Specifically, the flatband potential ranges from 1 to 1.2 V, while the acceptor density ranges from 2 × 10^18^ to 1.4 × 10^19^ cm^−3^. Moreover, the annealing temperature of 1000 °C resulted in the lowest resistance to charge transfer (Rct).

## Data Availability

The datasets used and/or analysed during the current study available from the corresponding author on reasonable request.
